# Rehabilitation Approach for Children With Joubert Syndrome and Related Disorders

**DOI:** 10.7759/cureus.38658

**Published:** 2023-05-07

**Authors:** Hiroshi Mano, Kenichi Kitamura, Mayumi Tachibana, Ai Suzuki, Toyohiro Yamauchi, Tomomi Murakami, Yoshinori Okumura, Masashi Koyama, Kenji Shimizu

**Affiliations:** 1 Department of Rehabilitation Medicine, Shizuoka Children's Hospital, Shizuoka, JPN; 2 Department of General Pediatrics, Shizuoka Children's Hospital, Shizuoka, JPN; 3 Department of Pediatric Neurology, Shizuoka Children's Hospital, Shizuoka, JPN; 4 Department of Radiology, Shizuoka Children's Hospital, Shizuoka, JPN; 5 Department of Clinical Genetics and Cytogenetics, Shizuoka Children's Hospital, Shizuoka, JPN

**Keywords:** rehabilitation, orthotics, joubert syndrome and related diseases, joubert syndrome, developmental support

## Abstract

Joubert syndrome and related disorders (JSRD) are rare and intractable diseases characterized by delayed psychomotor development, hypotonia and/or ataxia, and abnormal respiratory and eye movements. Cerebellar vermis agenesis and molar tooth signs are distinct on cerebral magnetic resonance imaging (MRI). Children with JSRD present with delayed psychomotor development, including intellectual disability and emotional or behavioral problems. Rehabilitation treatments are provided to promote psychomotor development. However, limited reports and evidence exist on rehabilitation treatments for children with JSRD. Three children with JSRD received rehabilitation treatment. The children received rehabilitation treatment once a week to once every one to two months at our hospital and/or other facilities. All patients received physical, occupational, and speech-language-hearing therapy, depending on their symptoms and conditions. In children with tracheostomies due to abnormal respiration, respiratory physical therapy and speech-language-hearing therapy, including augmentative and alternative communication, were needed. For hypotonia and ataxia, an orthotic intervention was considered in all three cases, and foot or ankle-foot orthoses were used in two cases. Although there is no specific or established rehabilitation method for children with JSRD, appropriate rehabilitation approaches, including physical, occupational, speech-language-hearing therapies and orthotic intervention, should be considered and provided to improve their function and expand their activity and participation. Orthotic intervention for hypotonia seems reasonable for improving gross motor development and function in children with JSRD.

## Introduction

Joubert syndrome (JS) is a rare and intractable disease characterized by the agenesis of the cerebellar vermis, episodic hyperpnea, abnormal eye movements, ataxia, and psychomotor delay [1]. Various JS-related diseases, such as Senior-Loken syndrome [2,3], cerebellar vermis hypo/aplasia, oligophrenia, congenital ataxia, coloboma, hepatic fibrocirrhosis (COACH) syndrome [4], and Dekabann syndrome [5], have been reported previously. As a group of these diseases that show “Molar tooth sign” on cerebral magnetic resonance imaging (MRI), the concept of Joubert syndrome and related disorders (JSRD) has been proposed [6]. Arima syndrome is considered a severe form of JSRD with symptoms in the kidney, retina, and liver [7,8]. JSRD is classified as a ciliopathy, and various causative genes have been identified [9]. Children with JSRD present psychomotor developmental delays, including intellectual disability and emotional and behavioral problems [10]. However, it is unclear which rehabilitation interventions are needed to promote development and improve the function of children with JSRD. There are very few reports on rehabilitation interventions for JSRD [11].

## Case presentation

This case presentation was approved by the Ethics Committee of Shizuoka Children’s Hospital (ethical approval number: R4-94). Written consent was obtained from the guardians for the presentation of the cases. In accordance with the JSRD guidelines in Japan [11], all patients met the diagnostic criteria for JSRD but not for Arima syndrome. The "Molar tooth sign" image, which is characterized by a deep posterior interpeduncular fossa, thickened and elongated superior cerebellar peduncles, and hypoplasia or agenesis of the cerebellar vermis, was observed in all three children.

Child 1

**Figure 1:**
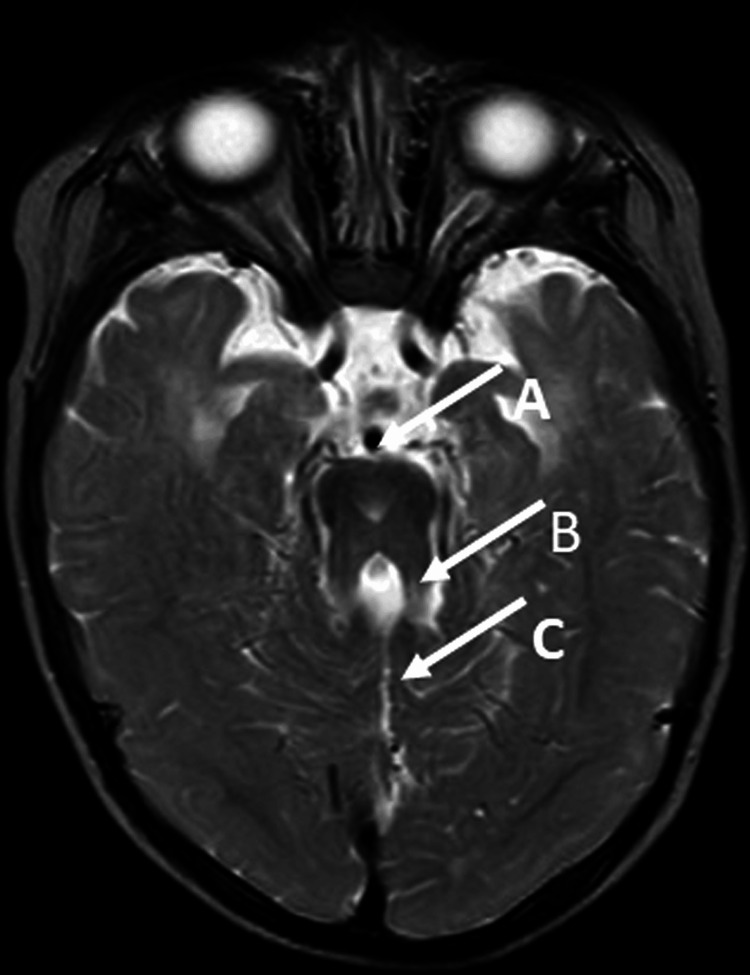
T2-weighted axial image on MRI of child 1 The MRI image was taken when she was 10 months old. The "Molar tooth sign" image is characterised by a deep posterior interpeduncular fossa (white arrow A), thickened and elongated superior cerebellar peduncles (white arrow B), and hypoplasia or agenesis of the cerebellar vermis (white arrow C). MRI: magnetic resonance imaging

The patient, a three-year-old girl, was born at full term and had a normal birth weight. At four months of age, she was referred to the plastic surgery department of our hospital for polydactyly of the left upper limb (the excess finger was surgically removed at one year of age). At nine months, she was referred to the Pediatric Neurology Department for developmental delay. At 10 months, she visited the rehabilitation department, and a rehabilitation intervention was initiated to promote motor development. In the same month, she underwent a cerebral MRI and was diagnosed with JS. The MRI image is shown in Figure 1.

**Figure 2:**
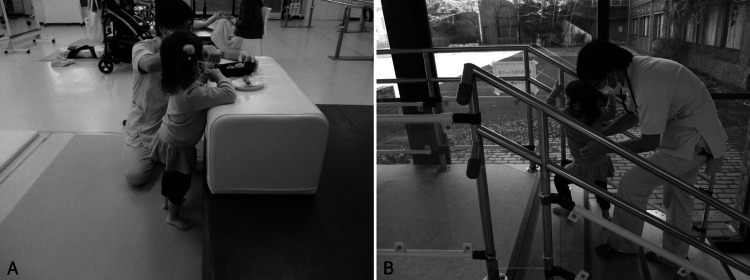
Situations during rehabilitation therapy: Child 1 (A) She stands alone by holding onto something. She plays in the house while standing. (B) She was willing to walk and climb the stairs. Because she could not walk alone at that time, the physical therapist supported her posture lightly.

During rehabilitation, physical therapy was administered to achieve milestones of motor development. Physical therapy was provided once a week in conjunction with a nearby hospital in her regional area. She had been prescribed a foot orthosis for hypotonia and ataxia to stabilize her ankle joints while standing and has been using it since age two. Her motor developmental milestones were as follows: head control at eight months, rolling over at 13 months, and sitting up/sitting alone at 20 months. She trained stand, walk, and climb stairs with assistance (Figures 2A, 2B) and could stand and walk alone at 45 months of age. Her main means of moving into the room was crawling or walking. When moving indoors and outdoors, she is generally transported in a caregiver's arms or a stroller. The first word was used at 28 months of age as a cognitive and language developmental milestone. Nonverbal expressions, such as pointing, were also observed. In addition to continuing the physical therapy, we are considering initiating speech-language-hearing therapy to improve her cognitive and language development. The patient did not present with abnormal respiration or require respiratory physical therapy. No hand dysfunction due to polydactyly or surgery was observed.

Child 2

**Figure 3:**
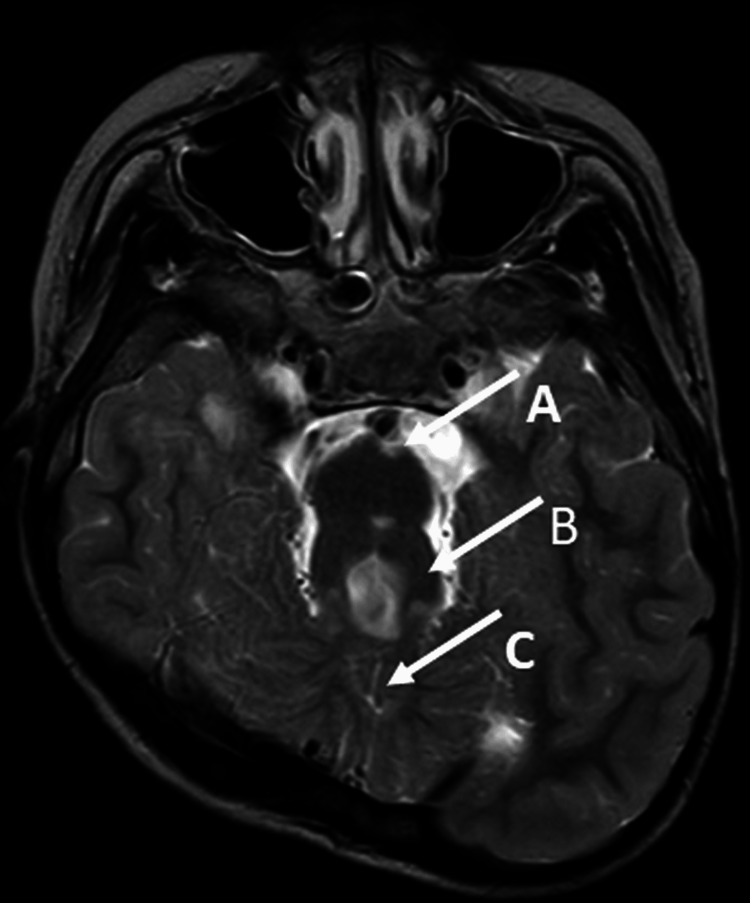
T2-weighted axial images on MRI of child 2 The MRI image was taken when he was nine years of age. The "Molar tooth sign" image is characterised by a deep posterior interpeduncular fossa (white arrow A), thickened and elongated superior cerebellar peduncles (white arrow B), and hypoplasia or agenesis of the cerebellar vermis (white arrow C). MRI: magnetic resonance imaging

The patient, 11-year-old boy, was born at full term and had a normal birth weight. At nine months of age, he was referred to the Pediatric Neurology Department of our hospital for a second opinion on JS (already diagnosed at a previous hospital), especially respiratory management. In the same month, he underwent a tracheostomy and was started on mechanical ventilation at our hospital. He was discharged from our hospital, and home mechanical ventilation therapy was initiated. He underwent gastrostomy at the age of two. Despite multiple hospitalizations yearly due to respiratory infections, he was weaned off mechanical ventilation at the age of four. In the same year, he underwent fundoplication for gastroesophageal reflux. He has not been hospitalized for respiratory infections since then. The patient continued to receive home oxygen therapy. The MRI image is shown in Figure 3.

**Figure 4:**
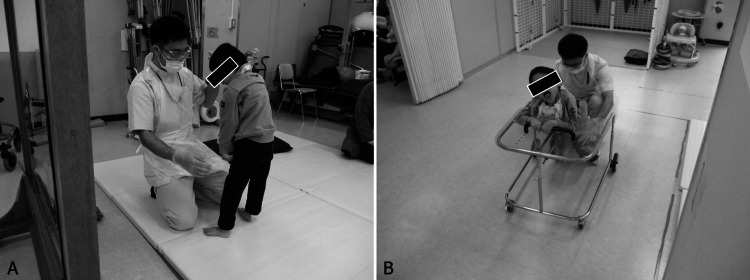
Situations during rehabilitation therapy: Child 2 (A) He could hold a standing position with one hand, assisted by a physical therapist. (B) He practiced ambulation using a walker with a saddle.

Respiratory physical therapy was the primary rehabilitation treatment. After four years of age, physical and occupational therapies were administered once every one to two months to improve motor and cognitive functions. His motor developmental milestones were as follows: head control at age three, rolling over at age three, and sitting up/sitting alone at age eight. He was prescribed a plastic ankle foot orthosis to stabilize his ankle joints during standing and walking training at the age of seven. He required assistance in standing and walking and is currently undergoing training (Figures 4A, 4B). When moving indoors or outdoors, he is generally transported in attendant-controlled manual wheelchairs with modular seating systems. An evaluation by the respiratory support team at our hospital determined that weaning from tracheostomy and home oxygen therapy will continue to be difficult. However, the patient no longer required frequent respiratory physical therapy. He could not express himself verbally because of severe developmental delays or tracheotomy. He expresses his "No" intention by shaking his head, but his expression of "Yes" intention is unclear. The patient attended a special support school. He is currently receiving rehabilitation, mainly at a regional hospital, because our hospital is far from his hometown, and his medical condition is stable.

Child 3

**Figure 5:**
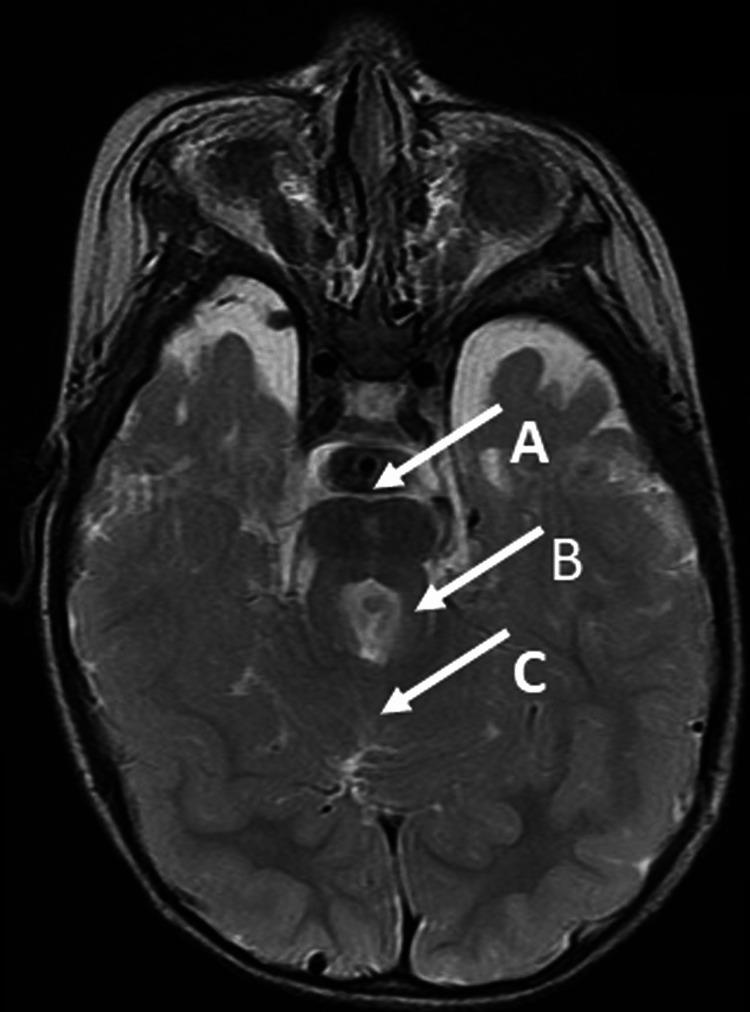
T2-weighted axial images on MRI of child 3 The MRI image was taken when he was 21 months old. The "Molar tooth sign" image is characterised by a deep posterior interpeduncular fossa (white arrow A), thickened and elongated superior cerebellar peduncles (white arrow B), and hypoplasia or agenesis of the cerebellar vermis (white arrow C). MRI: magnetic resonance imaging

The patient, 13-year-old boy, was born at full term and had a normal birth weight. At three months of age, he was referred to the Clinical Genetics and Cytogenetics Department of our hospital with suspected JS. In the same month, he underwent a tracheostomy and was started on mechanical ventilation at our hospital for a central apnea episode. Thereafter, the patient was managed at the referring hospital, and a definitive diagnosis of JS was made at another university hospital with expertise in the syndrome. He was referred to our hospital for respiratory and nutritional management, and he underwent gastrostomy at the age of four. The patient was weaned off the mechanical ventilator at age nine. The patient was not hospitalized for any respiratory infections. The patient continued to receive home oxygen therapy. The MRI images are shown in Figure 5.

**Figure 6:**
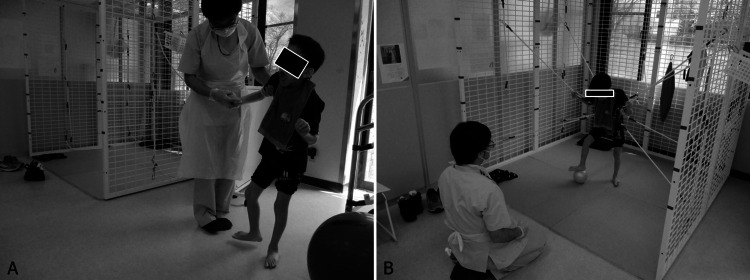
Situations during rehabilitation therapy: Child 3 (A) He could walk with assistance from his hands. The image was processed to hide the eyes in order to remove information that would identify the patient. (B) He practised soccer with postural support and load reduction.

He attended a regional rehabilitation facility for children with disabilities and used short-term hospitalization for intensive rehabilitation at a distant pediatric rehabilitation facility (physical, occupational, and speech-language-hearing therapies were provided); rehabilitation treatment was not provided at our hospital. As it had become difficult to continue attending other hospitals for rehabilitation, his guardians wanted to receive rehabilitation therapy at our hospital at the age of 10 years. With assistance, the patient was able to stand and walk. Although the tracheostomy cannula was not a speech cannula, the patient could generate a voice. No significant words were identified. He received physical therapy through a home-visit rehabilitation service once or twice a month. At our hospital, low-frequency physical and speech-language-hearing therapies are initiated every three to six months. In physical therapy, the prescription of ankle-foot orthotics was considered for the ankle joint and foot instability due to valgus foot. However, because of the improved stability due to increased muscle strength, wearing a chukka or high-cut shoes was chosen instead. In speech-language-hearing therapy, tablet devices are considered augmentative and alternative communication. However, he could express three-word sentences equivalent by gestures, and using this device was impractical. He attended a special support school and used a walker to ambulate or pedal a wheelchair to move around. The practical means of indoor and outdoor transportation is hands-in-hand walking. He was also involved in sports, such as soccer and boccia (Figures 6A, 6B). Pediatric surgeons at our hospital determined that weaning from tracheostomy and home oxygen therapy will continue to be difficult because of glossoptosis. However, there is little need for respiratory therapy.

The clinical features of three patients with JSRD are shown in Table 1.

**Table 1:** Clinical features of patients in this report ^a^ Diagnosis criteria of Joubert Syndrome and Related Disorders: Definite: A-1, A-2, and B-1 are all satisfied, and C is ruled out. Probable: A-1, A-2, A-3, and B-2 are all satisfied, and C is ruled out, or A-1, A-2, A-4, and B-2 are all satisfied, and C is ruled out. ^b^ MRI: magnetic resonance imaging ^c^ The developmental quotient is calculated by dividing the developmental age by the calendar age and multiplying by 100. ^d^ Vineland-II: Vineland Adaptive Behavior Scales, Second Edition ^e^ The standard scores have a mean of 100 and a standard deviation of 15. The lower limit of the scores is 20. For those seven years of age or older, motor skills score is the estimated derived score. ^f^ The standard scores have a mean of 15 and a standard deviation of 3.

Patients			Child 1	Child 2	Child 3
Age, years			3	11	13
Sex			Female	Male	Male
Diagnosis criteria ^a^	A-1.	Psychomoter development delay	+	+	+
	A-2.	Hypotonia and/or ataxia (present or previous)	+	+	+
	A-3.	Abnormal respiratory (present or previous)	-	+	+
	A-4.	Abnormal eye movement	+ (nystagmus, strabismus)	+ (strabismus)	+ (strabismus)
	B-1.	Molar tooth sign on MRI ^b^	+	+	+
	B-2.	Cerebellar vermis agenesis without Molar tooth sign	not applicable	not applicable	not applicable
	C	Differential diseases: Arnold Chiari Malformation, Dandy-Walker syndrome, Cogan's syndrome, Hereditary and isolated cerebellar dysplasia, arachnoid cyst, spinocerebellar degeneration	ruled out	ruled out	ruled out
Associated symptoms	Eye/Retina	Retinopathy, Coloboma	-	+ (retinopathy)	-
	Respiratory	Tracheotomy	-	+	+
		Home Mechanical Ventilation	-	+/- (weaned at age 4 years)	+/- (weaned at age 9 years)
		Home Oxygen Therapy	-	+	+
	Kidney	Renal dysfunction, Polycystic kidney, Nephronophthisis	-	-	-
	Liver	Impairment of liver function, Hepatofibrosis	-	-	-
	Limb/Extremity	Polydactyly	+	+	-
Genetic testing		Detection of causative genes	-	+ (TCTN2 gene)	-
Developmental milestones	Motor	Head control	8 months	3 years	< 3 years
		Rolling over	13 months	3 years	< 3 years
		Sitting up/Sitting alone	20 months	8 years	3 years
		Standing up/Standing alone	45 months	- (standing with assistance)	- (pulling oneself up and standing alone holding onto something)
		Walking alone	45 months	- (walking with assistance)	- (walking with assistance)
	Speech	First word	28 months	- (expression of "No" by shaking head)	- (expression equivalent to 3-word sentences with gestures)
		Two-word phrase	-	-	-
Developmental/Behavioural assessment	Enjoji scale	Age at examination, years	3	11	13
		Developmental quotient ^c^	54	3	12
		Exercise domain ^c^	41	6	6
		Sociality domain ^c^	59	2	12
		Language domain ^c^	63	3	16
	Vineland-II ^d^	Age at examination, years	3	11	13
		Adaptive behaviour composite ^e^	55	20	20
		Communication ^e^	65	20	20
		Daily Living Skills ^e^	63	20	20
		Socialization ^e^	51	20	20
		Motor Skills ^e^	51	(20)	(20)
		Maladaptive behaviour index ^f^	17	17	21

## Discussion

There are very few reports on rehabilitation treatment for children with JSRD. Dekair et al. reported a rehabilitation treatment program for a girl with JS focusing on improving the child’s motor, language, cognitive, and social skills, including physical, occupational, and speech-language-hearing therapies; orthotic services improved her development but continued to be delayed for her age [12]. Regarding orthotic interventions, there have been reports of the prescription and use of a stander [13] and ankle-foot orthosis [14]. For hypotonia and ataxia, it is reasonable to improve the stability and limit the degrees of freedom of the lower limbs. Orthoses were considered in all three of our cases. Orthotic therapy should be considered to improve gross motor development and function in children with JSRD.

In JS patients, while psychomotor developmental delay and emotional or behavioral problems vary, a positive correlation between age and equivalent age on cognitive and adaptive behavior tests has been reported, indicating that children with JSRD can continue to develop gradually and acquire new skills until adulthood [10]. According to children's symptoms and developmental stages, appropriate rehabilitation approaches, including physical, occupational, speech-language-hearing therapies and orthotic intervention, should be considered and provided to expand their activity and participation.

## Conclusions

Although there is no specific or established rehabilitation method for children with JSRD, appropriate rehabilitation approaches should be considered and provided to improve their function and expand their activity and participation. Orthotic intervention for hypotonia seems reasonable for improving gross motor development and function in children with JSRD.
